# Variability of the Ionome of Wild Boar (*Sus scrofa*) and Red Deer (*Cervus elaphus*) in a Dutch National Park, with Implications for Biomonitoring

**DOI:** 10.1007/s12011-023-03879-7

**Published:** 2023-10-09

**Authors:** Elke Wenting, Henk Siepel, Patrick A. Jansen

**Affiliations:** 1 Department of Environmental Sciences, Wageningen University and Research, Box 47, 6700 AA Wageningen, the Netherlands; 2 Radboud Institute for Biological and Environmental Sciences, Department of Animal Ecology and Physiology, Radboud University, Box 9010, 6500 GL Nijmegen, the Netherlands; 3 Smithsonian Tropical Research Institute, Balboa, Ancon, Panama

**Keywords:** Ecological stoichiometry, Ionomics, Minerals, Trace elements, Macro nutrients, Heavy metals

## Abstract

The ionome—an important expression of the physiological state of organisms—is poorly known for mammals. The focus on particular tissues—such as liver, kidney, and bones—in biomonitoring of environmental pollution and potential deficiencies is based on widely held assumptions rather than solid knowledge of full mammalian ionomes. We examined the full ionome of Red deer (*Cervus elaphus*) and Wild boar (*Sus scrofa*), two commonly used mammals for biomonitoring, in a Dutch protected nature reserve (Veluwezoom). We used four individuals per species. We dissected 13 tissues and organs from each individuals (eight in total) of each species and measured 22 elemental concentrations in each. We assessed, for each element, how concentrations varied across tissues within and between individuals. Based on existing literature, we put our findings in the context of their function in the mammalian body. We found that the ionome was highly variable between as well as within the two species. For most elements, tissues containing the highest and lowest concentration differed between individuals. No single tissue accurately represented the accumulation of toxic elements or potential deficiencies in the bodies. Our assessment of the element’s biological roles revealed a serious lack of reference values. Our findings imply that analyses of commonly used tissues in biomonitoring do not necessarily capture bioaccumulation of toxins or potential deficiencies. We recommend establishing a centralized database of mammalian ionomes to derive reference values in future. To our knowledge, our study is one of the most complete assessments of mammalian ionomes to date.

**Supplementary Information:**

The online version contains supplementary material available at 10.1007/s12011-023-03879-7.

## Introduction

The elemental composition of organisms—the ionome—is an important expression of their physiological state [1, 2]. Like ecological stoichiometry—the study of the balance of energy and chemical elements in ecological interactions [3–5] -, ionomics has been applied to population ecology to understand allocation and life history plasticity [6, 7] and population growth [8, 9]. The ionome relates to a wide variety of ecological processes, including foraging, scavenging and carrion decomposition [10, 11]. So far, ionomics and ecological stoichiometry have been widely applied to plants [e.g. 12–15], invertebrates [e.g. 16, 17], and fish [e.g. 18, 19]. Insights into the mammalian ionome, particularly large mammals, are limited.

The ionome reflects both the nutrient status and ecotoxic load of animals. While foraging across landscapes, large mammals accumulate a wide range of elemental nutrients in their bodies over their lifespan [20]. These include essential trace elements—e.g. cobalt (Co) and selenium (Se)—that are hard to gather for animals in sufficient amounts, especially in nutrient-poor areas. Large mammals may experience difficulties in acquiring sufficient amounts of essential elements, increasing the risk of deficiencies. This particularly applies to areas with unbalanced nutrient availability, for example due to decades of high nitrogen (N) deposition, which leads to acidified soils and leaching of cations to deeper horizons, hence increasing the risks of deficiencies for wildlife [21–23].

While foraging, large mammals may also take up potential ecotoxic elements, such as arsenic (As), cadmium (Cd) and lead (Pb). Large mammals, especially ungulates that are the main consumers of vegetation [e.g. 24], are therefore commonly used for biomonitoring of such ecotoxic elements [25]. Biomonitoring of environmental pollution—i.e. ecotoxicity—is mainly based on the assumption that ecotoxic elements would accumulate in liver, kidney, bones or hairs of wildlife, in order to be excreted from the body as fast as possible [e.g. 26–28]. These tissues are also most frequently used for assessing risks of elemental deficiencies of scarce elements [e.g. 29, 30]. However, it is uncertain that this assumption holds given the high variation of the mammalian ionome [e.g. 11, 31].

In Europe, ecotoxicity and potential deficiencies are commonly monitored by screening of particular tissues—mainly liver and kidney—of culled Red deer (*Cervus elaphus*) and Wild boar (*Sus scrofa*) [e.g.^32^–^37^]. For example, Nowakowska et al. [38] used liver and kidney samples of Wild boar to examine environmental levels of Se in Poland. Vikøren et al. [39] used the liver as an indication for the cupper (Cu), Co and Se concentration in wild Red deer in Norway. However, due to the lack of knowledge of the full mammalian ionome, it is uncertain whether liver and kidney are indeed the proper focal tissues for ecotoxic and scarce elements. The lack of extensive overviews of the full mammalian ionome also limit the interpretation of current ionomic studies [40].

For both ecotoxic and scarce elements, the distribution over the mammalian body thus remains poorly known and extensive overviews are missing [e.g. 41]. Most ionomic studies thus use an incomplete approach, which could unintendedly cause severe bias in the assessment of ecotoxicology and potential deficiencies in ionomic studies dealing with large mammals. Also, a systematic overview of the biological relevance of chemical elements for the mammalian body, as well as signs of deficiency or toxicity, is missing. This is needed to put existing and new ionomic insights in the context of their biological and physiological role.

Here, we aimed to gain more insight in the ionome of large mammals, particularly in how elements are distributed across the body. We measured the concentrations of 22 elements in 13 different tissues from four individuals of Red deer and four individuals of Wild boar (henceforth ‘deer’ and ‘boar’). These individuals were culled in a national park in The Netherlands, a mineral- and nutrient-poor environment that experienced decades of high N deposition, where deficiencies are likely to occur. We also tried to put our findings in the context of their function in the mammalian body, based on the literature.

## Methods

### Study Site and Species

We focused on deer and boar in Veluwezoom National Park (henceforth ‘Veluwezoom’), the Netherlands (52°02’N, 6°01’E), a protected area of 5,000 ha situated on partly glacier deposits and partly on cover sands over these deposits, making the natural mineral availability limited to very scarce (mineral-poor cover sands). Veluwezoom is a former agro-silvopastoral landscape that became a national park in 1930 [42]. It contains a mosaic of dry grass-heathlands, pastures, abandoned crop fields, and woodland, grazed by free-ranging Scottish highland cattle (*Bos taurus*) and Icelandic horses (*Equus ferus caballus*)—introduced in the 1980s—as well as by Roe deer (*Capreolus capreolus*), Fallow deer (*Dama dama*), Red deer and Wild boar [43]. Due to surrounding highways former pollution (especially before 1980’s) with Pb might be possible. Samecka-Cymerman et al. [44] found elevated levels of Pb in bryophytes collected from Veluwezoom.

The Red deer is a herbivorous ruminant ungulate that is associated with woodland habitats [e.g. 45, 46]. Only the males have antlers, that cast in spring and regrow in late summer [e.g. 47]. Single-born calves are born in spring. The mating season (“rut”), which costs lots of energy, is from September to November with a peak in October [e.g. 48, 49].

The Wild boar is a monogastric, opportunistic omnivorous ungulate that is known for its highly plastic diet and their ability to adapt to diverse food and habitats [e.g. 50]. Reproduction is less seasonal and litter sizes range from one to twelve piglets, depending on many factors including the maternal body weight and summer temperature [e.g. 51].

### Carcass Dissection

We used freshly culled carcasses that were obtained in the culling season 2019–2020 (culled between October 2019 and March 2020), and used four individuals of each species. Local hunters used Pb free RWS HIT ammunition. Culling at Veluwezoom is only used as an intervention against wildlife damage, i.e. culled individuals are not harvested but left to decompose in nature. For deer, we used two young females (‘RD1’ and ‘RD2’), one young male (‘RD3’), and one male calf (‘RD4’). For boar, we used two male piglets (‘WB1’ and ‘WB2’) and two female piglets (‘WB3’ and ‘WB4’). For each carcass, we dissected 13 tissues belonging to different organ systems [52]: bone; skin and hair; muscle; brain; eyes; lungs; heart; spleen; kidney; liver; pancreas; stomach (including rumen for deer); and intestines. We were able to dissect all these tissues from all the carcasses, except the pancreas for the male calf (RD4) and a male piglet (WB1).

No animals were killed for the purpose of our study. According to the Animal Welfare Officer of Wageningen University & Research, our study is not considered as experimentation on animals (Appendix 1), and therefore permitted under Dutch law.

The dissection procedure was performed in two steps. First, we dissected seven of the tissues—skin and hair, muscle, lungs, heart, spleen, kidney, and liver—in a self-made dissection room at Veluwezoom. We also collected the entire guts, head and the right hind leg that we needed to create tissue samples for the other six tissues. The carcass remains after dissection were returned to nature. Second, we further dissected the guts—pancreas, stomach, and intestines -, the head—brain and eyes -, and the hind leg—bone—in the dissection room of Wageningen Environmental Research. For the bone, we sawed a piece of bone from the lower leg, that we cleaned by boiling it a few minutes. Once dissected, we stored all the collected tissues in the freezer at minus 18 °C until we further processed them into homogeneous tissue samples.

For culling purposes only, the local game wardens occasionally provide mineral licks for the deer and corn for the boar. We analyzed these mineral licks—two different types—and the corn using the same procedure as described below since mineral licks and additional feeding might help ungulates to compensate for any deficiencies in their diet [e.g. 53]. However, since we can only speculate about the consequences for the ionome of deer and boar that we analyzed, we do not discuss the elemental composition of these salt licks and corn (Appendix 2), and potential effects on the ionome of deer and boar, in detail in this study.

### Measurements

Each collected tissue was homogenized in the dissection room of Wageningen Environmental Research by grinding it with a blender. We stored about 15–25 g of the grinded tissue—three table spoons—in plastic bags. The tissue samples were frozen at minus 18 °C before we transferred them into plastic tubes for freeze-drying. The freeze-dried samples were transported to Radboud University, where we further prepared them for the chemical analysis.

We used a microwave digestion method with 5 mL 65% nitric acid (HNO_3_) and 2 mL 30% hydrogen peroxide (H_2_O_2_) to prepare the tissue samples for measuring the elemental concentrations with Inductively Coupled Plasma Optical Emission Spectroscopy (ICP-OES) and Inductively Coupled Plasma Mass Spectroscopy (ICP-MS). We measured 22 elemental concentrations for all the tissue samples. We used ICP-OES to measure 7 elements: calcium (Ca), potassium (K), magnesium (Mg), sodium (Na), phosphorus (P), sulfur (S), and silicon (Si). The other 15 elements were measured using ICP-MS: aluminum (Al), As, boron (B), Cd, Co, chromium (Cr), Cu, iron (Fe), manganese (Mn), molybdenum (Mo), nickel (Ni), Pb, Se, strontium (Sr), and zinc (Zn). We used the same devices as in Wenting et al. [11], meaning that the reported spike-and-recovery experiments also apply to this study. Correspondingly, the accuracy of these devices was guaranteed—besides using certified reference material for every microwave run—by using the following quality controls (QC): Multi element standard IV, Merck 1.11355; Phosphate standard, Merck 1.19898; Sulphate standard, Merck 1.19813; and Silicium standard, Merck 1.70236. The QC matrices were considered to correspond to the sample matrices since for both, any contamination of HNO_3_ and H_2_O_2_ was eliminated by using blanks (see for more details, including spike-and-recovery experiments, Wenting et al. [11]).

## Results

**Fig. 1:**
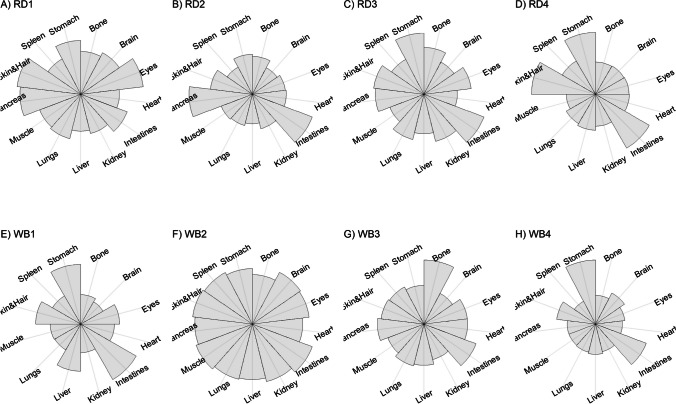
Distribution of aluminium (Al) per tissue per individual

**Fig. 2:**
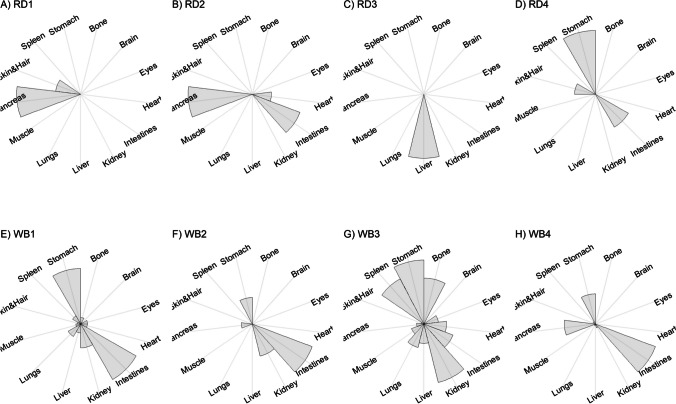
Distribution of arsenic (As) per tissue per individual

**Fig. 3:**
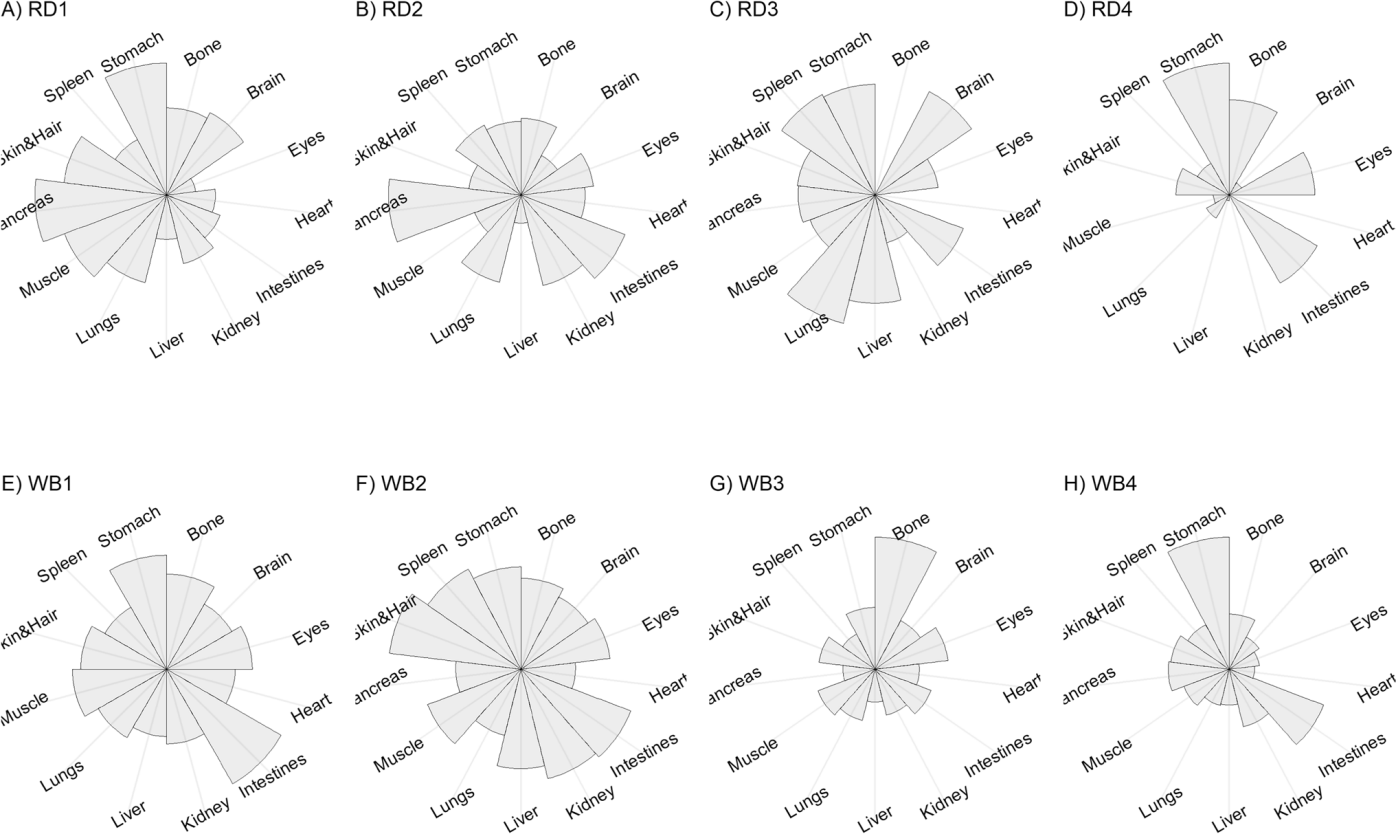
Distribution of boron (B) per tissue per individual

**Fig. 4:**
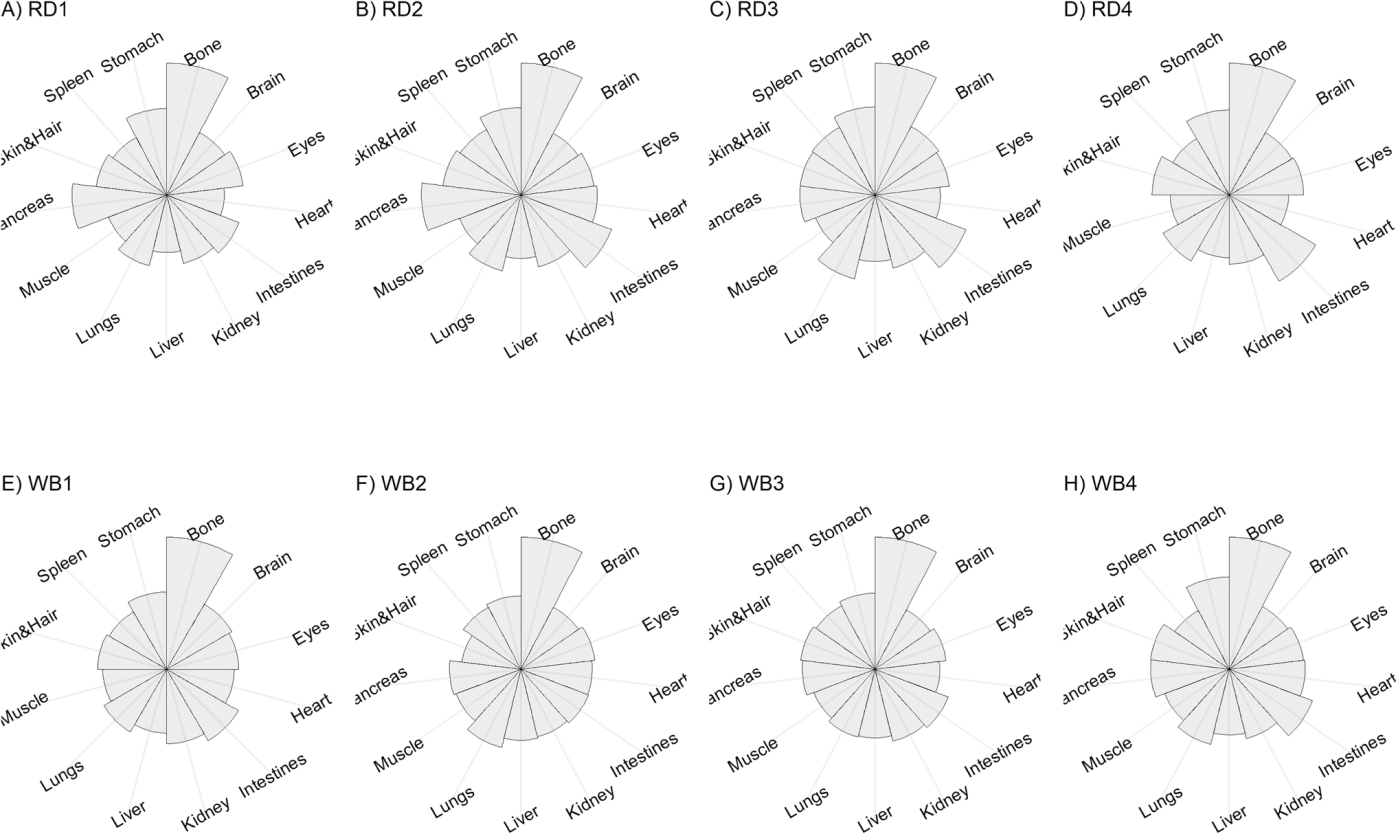
Distribution of calcium (Ca) per tissue per individual

**Fig. 5:**
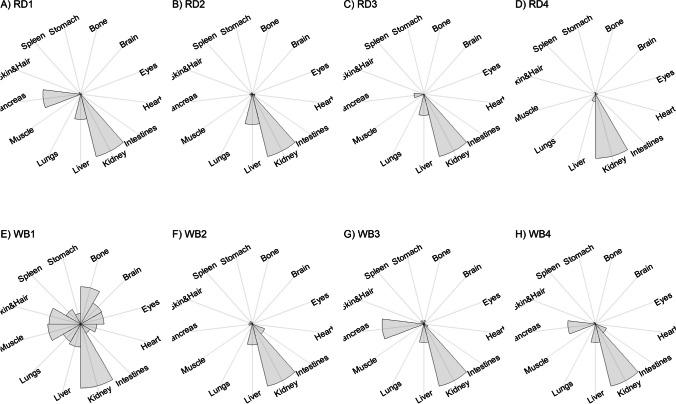
Distribution of cadmium (Cd) per tissue per individual

**Fig. 6:**
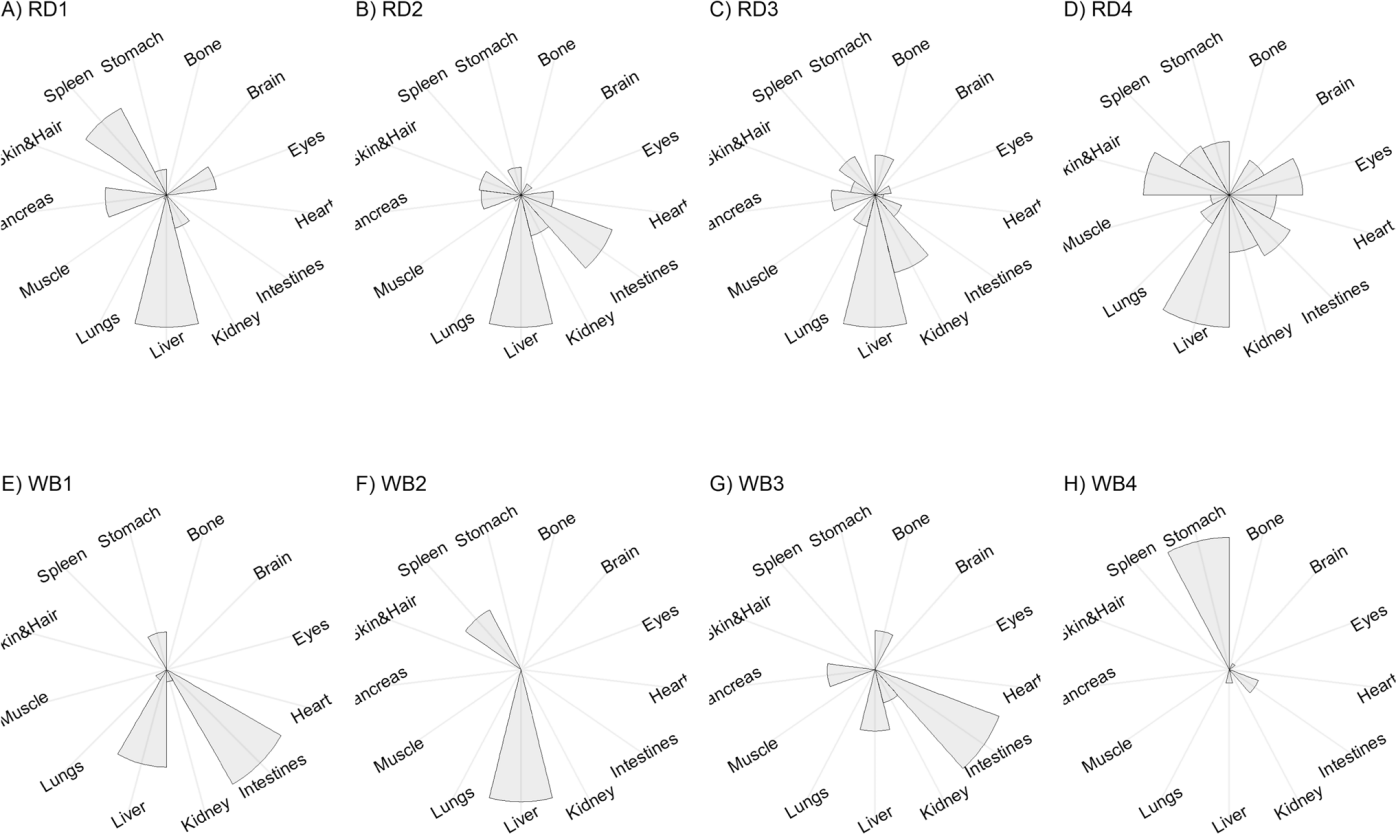
Distribution of cobalt (Co) per tissue per individual

**Fig. 7:**
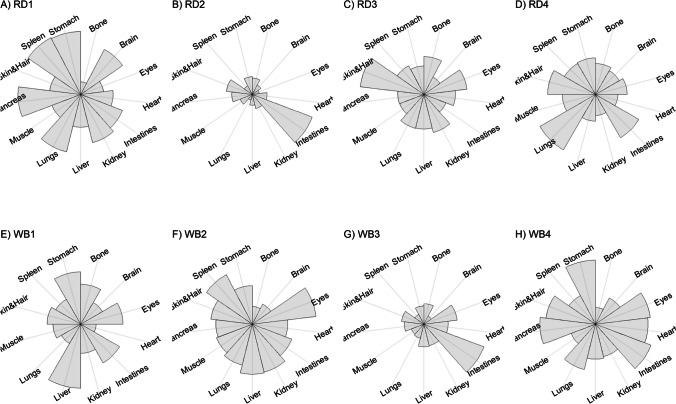
Distribution of chromium (Cr) per tissue per individual

**Fig. 8:**
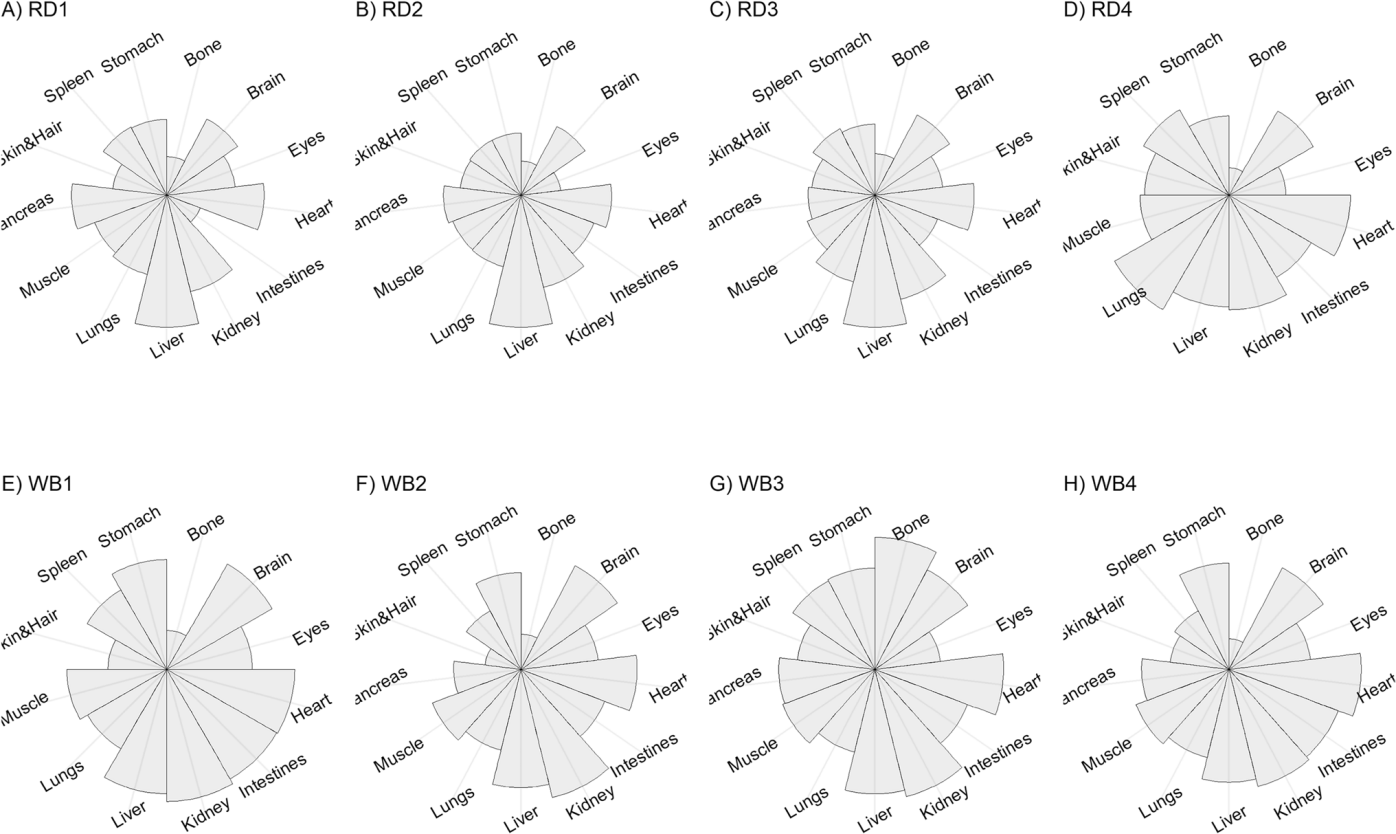
Distribution of copper (Cu) per tissue per individual

**Fig. 9:**
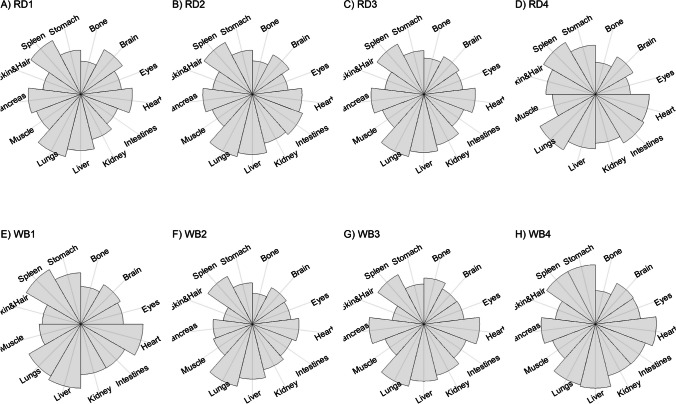
Distribution of iron (Fe) per tissue per individual

**Fig. 10:**
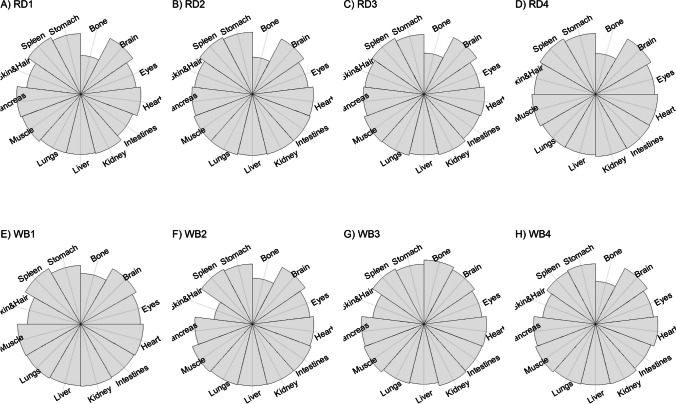
Distribution of potassium (K) per tissue per individual

**Fig. 11:**
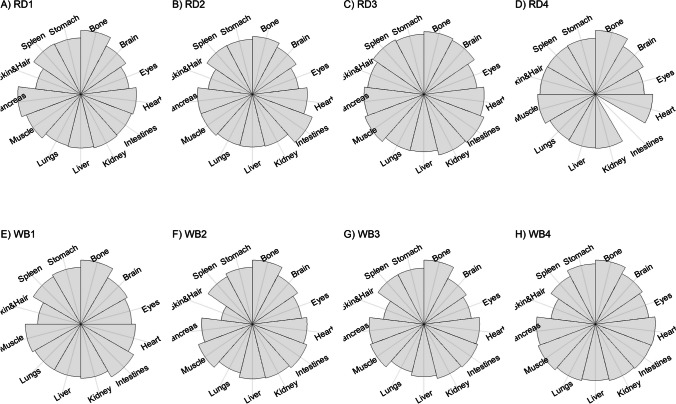
Distribution of magnesium (Mg) per tissue per individual

**Fig. 12:**
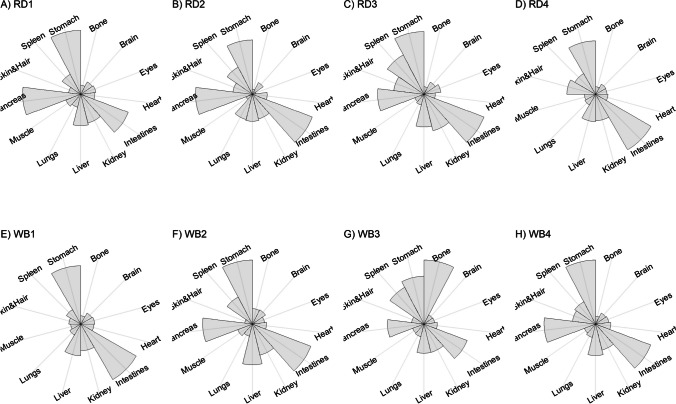
Distribution of manganese (Mn) per tissue per individual

**Fig. 13:**
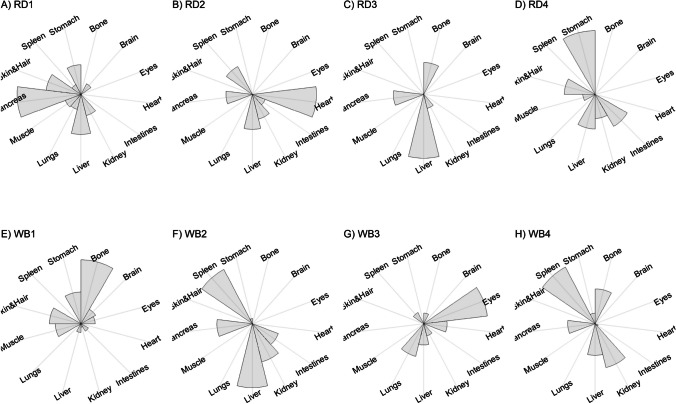
Distribution of molybdenum (Mo) per tissue per individual

**Fig. 14:**
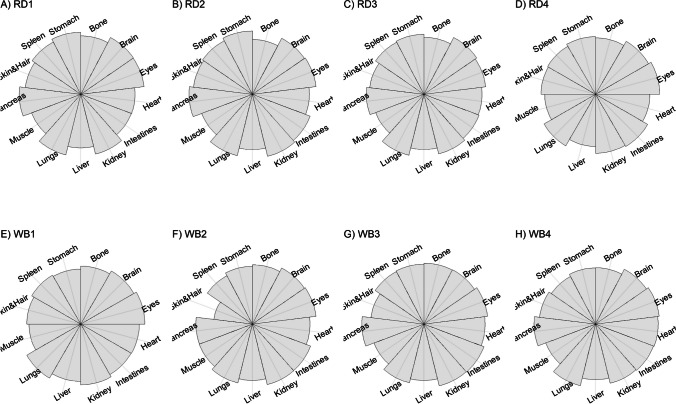
Distribution of sodium (Na) per tissue per individual

**Fig. 15:**
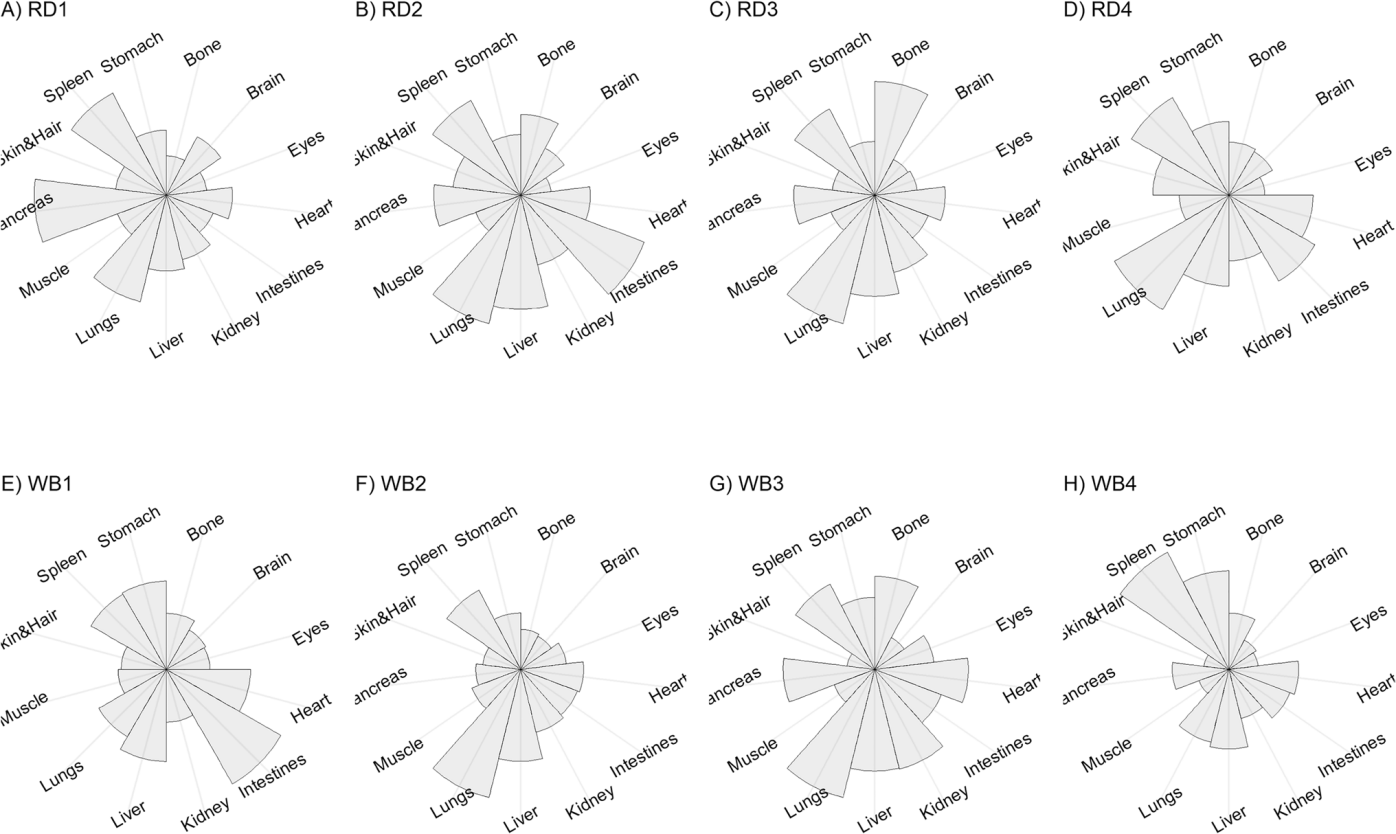
Distribution of nickel (Ni) per tissue per individual

**Fig. 16:**
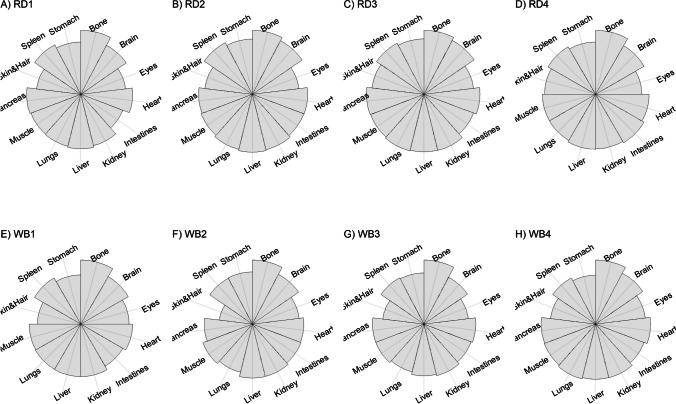
Distribution of phosphorous (P) per tissue per individual

**Fig. 17:**
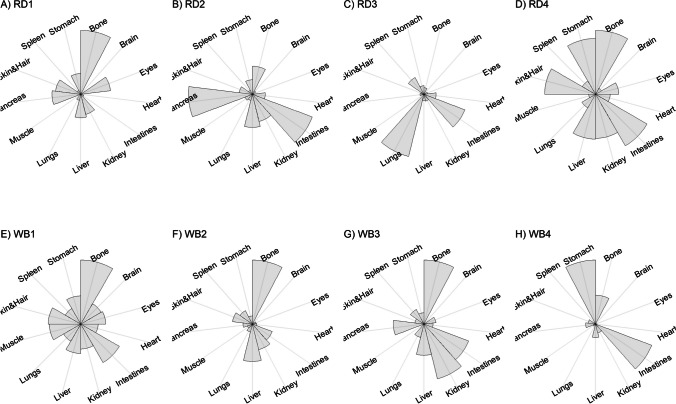
Distribution of lead (Pb) per tissue per individual

**Fig. 18:**
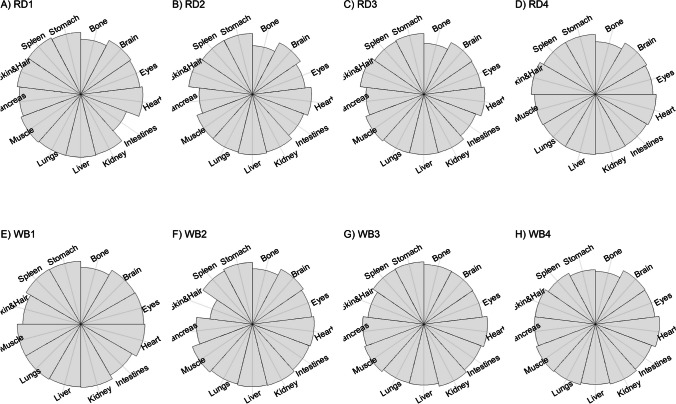
Distribution of sulfur (S) per tissue per individual

**Fig. 19:**
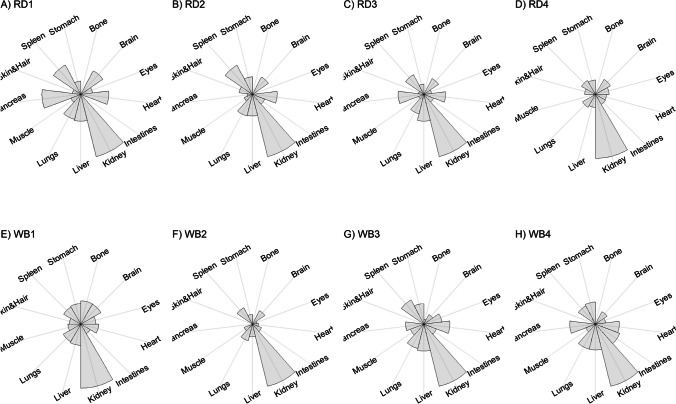
Distribution of selenium (Se) per tissue per individual

**Fig. 20:**
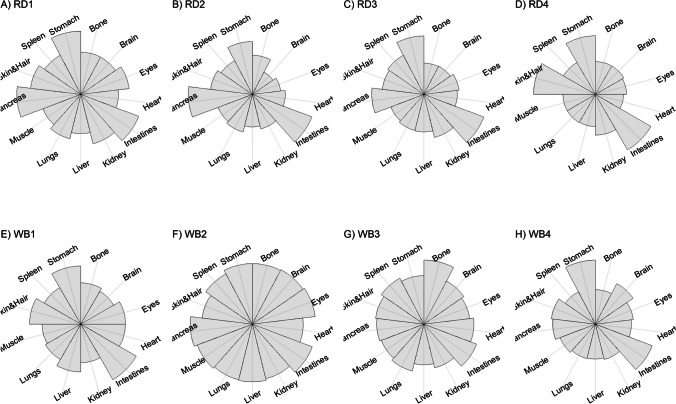
Distribution of silicon (Si) per tissue per individual

**Fig. 21:**
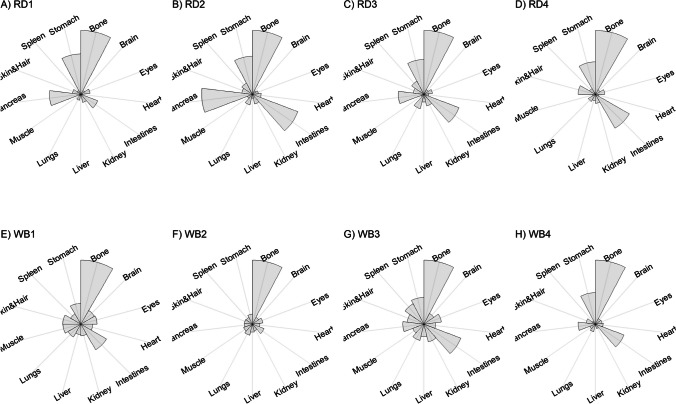
Distribution of strontiunm (Sr) per tissue per individual

**Fig. 22:**
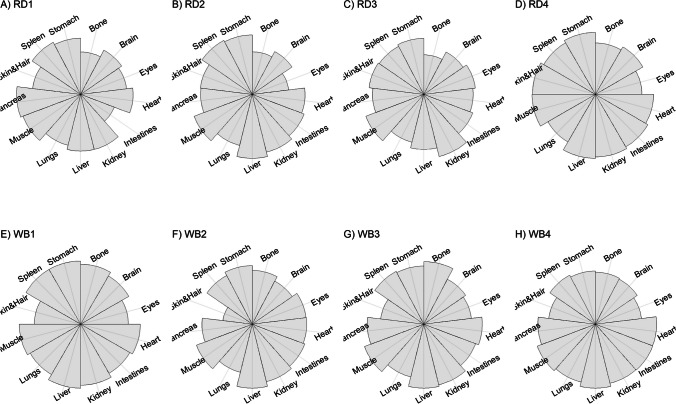
Distribution of zinc (Zn) per tissue per individual

We present our results in a descriptive way due to the low sample sizes that we used, with four individuals of each species. First, we summarized the total concentrations per element per individual in a table (Table 1). This table revealed variation in the total concentrations that we measured, which may indicate variation amongst tissues as well. Second, we listed the highest and lowest concentration measured per element for deer (Table 2) and for boar (Table 3), including the tissues in which these were found. For most elements, the tissues containing the highest and lowest concentrations varied within and between the species. Third, we used wind rose diagrams, with log(y + 1)-scale, to visualize how the total elemental concentration—as noticed in Table 1—is distribution over the 13 tissues (Figs. 1, 2, 3, 4, 5, 6, 7, 8, 9, 10, 11, 12, 13, 14, 15, 16, 17, 18, 19, 20, 21, 22).

**Table 1:** Total concentrations (µg Kg^−1^) of elements in individuals of red deer and wild boar collected from Veluwezoom National Park, the Netherlands. Elements are in alphabetical order

	RD1	RD2	RD3	RD4	WB1	WB2	WB3	WB4
Al	287	1,172	433	1165	895	393	526	1,141
As	0.220	0.290	0.010	0.430	24.07	0.800	12.06	26.66
B	32.51	88.37	32.87	16.02	75.24	43.46	65.20	55.65
Ca	351,232	171,542	155,282	302,348	435,317	348,739	469,061	256,532
Cd	27.25	39.98	15.08	5.12	19.57	15.19	27.30	20.54
Co	1.020	1.900	1.170	1.920	0.240	0.120	0.680	0.990
Cr	117.39	39.77	16.50	15.73	10.49	16.29	17.84	23.03
Cu	329.94	345.54	374.00	237.93	263.71	252.60	292.33	251.46
Fe	6,420	7,928	7,250	8,665	8,494	8,643	9,127	6,403
K	305,535	259,502	274,613	278,729	268,186	240,455	299,686	264,669
Mg	26,420	22,522	21,626	19,317	26,182	21,398	23,628	21,948
Mn	3,331	5,462	2,785	5,027	1,535	698	1,902	1,130
Mo	59.70	58.77	54.10	47.30	59.43	45.76	35.63	57.89
Na	152,728	145,511	149,330	156,760	139,680	117,545	146,317	158,860
Ni	105	85.02	72.92	77.50	146	80.14	92.23	111
P	376,183	273,821	286,736	356,541	399,327	344,298	223,948	320,518
Pb	4.67	14.00	619.94	13.61	24.89	4.82	9.01	21.41
S	193,954	174,096	182,296	187,548	169,640	144,988	170,016	168,743
Se	11.44	14.55	11.93	10.35	14.55	14.76	27.53	22.29
Si	1,768	3,054	1,362	2,391	1,911	1,407	1,797	3,153
Sr	163	106	78.71	107	182	234	27.18	129
Zn	2,207	1,822	2,418	1,972	1,667	1,939	1,938	1,671

**Table 2:** Highest and lowest concentrations (µg Kg-1), with corresponding tissue, in individuals of red deer collected from Veluwezoom National Park, the Netherlands

Element	RD1				RD2				RD3				RD4			
	Highest		Lowest		Highest		Lowest		Highest		Lowest		Highest		Lowest	
Al	50.92	Skin	8.75	Liver	450	Intestines	15.69	Liver	99.32	Intestines	10.02	Heart	366	Skin	13.72	Muscle
As	0.156	Pancreas	0.060	Skin	0.137	Pancreas	0.040	Heart	0.013	Liver	0.013	Liver	0.231	Stomach	0.070	Skin
B	5.50	Stomach	0.507	Eyes	24.34	Pancreas	0.995	Liver	5.66	Lungs	1.02	Kidney	5.21	Stomach	0.003	Kidney
Ca	329800	Bone	249	Liver	148300	Bone	302	Liver	138100	Bone	295	Muscle	279400	Bone	264	Muscle
Cd	19.80	Kidney	0.065	Eyes	34.78	Kidney	0.079	Stomach	13.13	Kidney	0.020	Spleen	4.77	Kidney	0.006	Intestines
Co	0.352	Liver	0.015	Lungs	0.643	Liver	0.027	Muscle	0.370	Liver	0.018	Heart	0.408	Liver	0.046	Muscle
Cr	20.01	Spleen	0.823	Bone	26.10	Intestines	0.353	Lungs	3.39	Skin	0.572	Brain	3.20	Lungs	0.599	Kidney
Cu	121	Liver	2.68	Intestines	156	Liver	2.67	Bone	137	Liver	3.690	Bone	51.39	Lungs	1.28	Bone
Fe	1944	Lungs	49.59	Bone	2394	Lungs	57.65	Bone	2362	Lungs	80.51	Bone	2678	Lungs	50.31	Bone
K	43000	Spleen	638	Bone	32980	Spleen	417	Bone	35220	Brain	844	Bone	34800	Spleen	29.40	Pancreas
Mg	5371	Bone	439	Skin	5735	Intestines	395	Skin	2581	Intestines	685	Eyes	4852	Bone	2.40	Pancreas
Mn	1887	Stomach	1.89	Bone	3133	Intestines	0.571	Eyes	1245	Intestines	1.334	Bone	3895	Intestines	1.71	Bone
Mo	34.09	Pancreas	0.053	Intestines	38.96	Heart	1.21	Intestines	42.16	Liver	0.057	Stomach	28.44	Stomach	0.919	Muscle
Na	22640	Brain	3694	Muscle	17440	Eyes	3805	Muscle	23590	Brain	3386	Muscle	33820	Eyes	3937	Muscle
Ni	31.37	Pancreas	1.81	Bone	17.35	Intestines	0.970	Eyes	18.64	Lungs	1.49	Brain	20.82	Lungs	1.30	Eyes
P	154900	Bone	3949	Skin	69890	Bone	3532	Eyes	65530	Bone	5561	Eyes	135000	Bone	5021	Eyes
Pb	1.65	Bone	0.029	Brain	4.28	Intestines	0.038	Eyes	535	Lungs	0.016	Brain	2.62	Bone	0.147	Muscle
S	21890	Skin	2002	Intestines	30890	Skin	2533	Bone	27680	Skin	3463	Bone	30520	Skin	19.70	Pancreas
Se	3.73	Kidney	0.227	Skin	6.44	Kidney	0.011	Bone	5.23	Kidney	0.170	Skin	5.76	Kidney	0.066	Muscle
Si	401	Pancreas	33.00	Heart	1135	Pancreas	14.60	Brain	507	Intestines	20.10	Bone	881	Intestines	18.70	Heart
Sr	125	Bone	0.116	Liver	50.27	Bone	0.267	Liver	50.87	Bone	0.194	Muscle	78.56	Bone	0.225	Muscle
Zn	408	Pancreas	28.30	Intestines	291	Liver	24.86	Eyes	464	Kidney	43.28	Bone	245	Liver	53.74	Eyes

**Table 3:** Highest and lowest concentrations (µg Kg^−1^), with corresponding tissue, in individuals of wild boar collected from Veluwezoom National Park, the Netherlands

Element	WB1				WB2				WB3				WB4			
	Highest		Lowest		Highest		Lowest		Highest		Lowest		Highest		Lowest	
Al	367	Intestines	9.64	Brain	45.31	Intestines	18.32	Bone	160	Bone	14.64	Brain	661	Stomach	12.61	Spleen
As	11.55	Intestines	0.070	Brain	0.400	Bone	0.060	Bone	2.71	Stomach	0.030	Brain	19.69	Intestines	0.010	Heart
B	16.65	Intestines	3.31	Liver	6.28	Skin&Hair	1.27	Heart	29.62	Bone	1.31	Pancreas	22.64	Stomach	0.830	Heart
Ca	421,300	Bone	523	Muscle	336,500	Bone	298	Skin&Hair	454,500	Bone	591	Heart	236700	Bone	478	Liver
Cd	6.97	Kidney	0.400	Intestines	12.76	Kidney	0.060	Bone	19.00	Kidney	0.020	Heart	16.03	Kidney	0.050	Heart
Co	0.110	Intestines	0.010	Kidney	0.080	Liver	0.040	Spleen	0.300	Intestines	0.070	Kidney	0.750	Stomach	0.010	Heart
Cr	1.97	Liver	0.300	Bone	2.41	Brain	0.400	Bone	6.95	Intestines	0.340	Brain	3.27	Stomach	0.46	Bone
Cu	42.82	Kidney	2.03	Bone	56.76	Kidney	1.95	Bone	43.97	Bone	5.68	Eyes	47.39	Heart	1.45	Bone
Fe	1996	Liver	83.82	Bone	4115	Lungs	43.1	Skin&Hair	2929	Lungs	51.43	Skin&Hair	1241	Liver	40.24	Bone
K	35310	Spleen	3916	Bone	31350	Brain	498	Skin&Hair	34200	Bone	4406	Skin&Hair	36270	Muscle	1068	Bone
Mg	6506	Bone	362	Skin&Hair	5118	Bone	65.1	Skin&Hair	5805	Bone	242	Skin&Hair	3485	Bone	295	Skin&Hair
Mn	953	Intestines	1.43	Bone	286	Stomach	1.00	Skin&Hair	1293	Bone	2.19	Brain	561	Stomach	1.20	Bone
Mo	40.75	Bone	0.120	Lungs	17.96	Liver	0.270	Spleen	24.48	Eyes	0.710	Bone	32.17	Spleen	0.190	Intestines
Na	25690	Eyes	3701	Muscle	18280	Eyes	376	Skin&Hair	18540	Eyes	3560	Skin&Hair	22810	Eyes	4245	Muscle
Ni	58.27	Intestines	2.81	Eyes	31.39	Lungs	1.67	Brain	22.22	Lungs	0.930	Skin&Hair	46.79	Spleen	1.10	Skin&Hair
P	200000	Bone	3787	Skin&Hair	155200	Bone	494	Skin&Hair	25780	Kidney	2791	Skin&Hair	110500	Bone	3711	Skin&Hair
Pb	7.74	Bone	0.800	Heart	1.76	Bone	0.030	Brain	2.41	Bone	0.190	Heart	9.48	Stomach	0.030	Brain
S	18780	Heart	6153	Bone	18720	Muscle	705	Skin&Hair	19950	Kidney	6064	Skin&Hair	22640	Heart	3861	Bone
Se	6.28	Kidney	0.400	Intestines	9.83	Kidney	0.180	Eyes	13.48	Kidney	0.040	Skin&Hair	10.12	Kidney	0.060	Liver
Si	686	Intestines	47.1	Brain	161	Intestines	54.4	Skin&Hair	485	Bone	49.70	Liver	1335	Stomach	47.10	Bone
Sr	150	Bone	1.24	Liver	220	Bone	0.800	Kidney	12.04	Bone	0.600	Heart	104	Bone	0.150	Liver
Zn	210	Liver	46.49	Skin&Hair	330	Liver	13.44	Skin&Hair	251	Liver	41.50	Skin&Hair	224	Liver	49.56	Eyes

As we aimed to put our findings in the context of their biological function, we report them per element separately, including a description of the biological relevance of each element for the mammalian body, as well as signs of toxicity and deficiency. We discuss the elements in alphabetical order. 

### Aluminum (Al)

Although Al is the third most common element in the earth’s crust, it is poorly absorbed in the animal body and there is no clear role described for this element [54, 55]. Due to long-lasting acidification of the environment due to excess N deposition mainly from agriculture, industry and traffic, pH values dropped significantly below 3.5 [22], which is much lower than the given pH value of 4.2 below which Al becomes soluble from soil aluminum oxides and hydroxides [56] and is thus potentially taken up by the vegetation, and so by animals. Although Al toxicity is associated with malabsorption of e.g. P (Allen 1984, cited in [54]), it remains unclear which and how other elements interplay with it to cause toxicity [54]. Most described symptoms of Al toxicity include inappropriately decreased feelings of thirst, refusal to swallow, and movement disorders such as hypokinesia [e.g. 57, 58].

Thurston et al. [59] and Pérez-Granados and Vaquero [55] described that Al will be mainly accumulated in bone when renal function is compromised. However, although the digestive organs tended to be important target tissues for some individuals (Fig. 1a-h; Tables 2 and 3), we found no particular tissue that contained most of the Al for the individuals we measured.

### Arsenic (As)

Contrary to Al, As is well absorbed but has no specific role in the animal body [54], although Frost et al. [60] described that As may function as antibiotic and anti-coccidial in swine and poultry. By our knowledge, it has not been used for that purpose in other animals [54]. Since it is well absorbed, As toxicity is likely to occur when food is contaminated with As [e.g. 61], for instance from Cu and Pb smelters [62]. When As exposure builds up slowly, animals may get used to it and ruminants may even develop a taste for it [63]. As appears to be most toxic in inorganic form [64, 65], and affects most organs, although kidney is mentioned as the most sensitive organ for As toxicity [66]. Commonly described symptoms of As toxicity include straining, abdominal pain, bone marrow depression with anemia, skin pigmentation changes, and diarrhea containing blood and mucus [e.g. 67–69].

Although the As concentration in WB2 was noticeable lower compared to the other boar, we found noticeably higher As concentrations in boar compared to deer (Table 1). It has been traditionally thought that As accumulates in the hairs [e.g. 70], however skin and hair did not contain the highest concentration for any individual (Fig. 2a-h; Tables 2 and 3). As was rather randomly scattered throughout the whole body for WB3 (Fig. 2g), and the guts appeared to be the main target tissues for the other individuals (Fig. 2a-h). We were not able to detect As in any other tissue than liver for RD3 (Fig. 2c; Table 2).

### Boron (B)

B is an essential trace element that is important for numerous life functions, including bone density, wound healing, embryonic development and metabolism of sex steroids and vitamin D (e.g. 71–73]. Although symptoms of B toxicity are poorly understood in animals [e.g. 74], acute B toxicity has been described for humans, including symptoms as nausea, vomiting, diarrhoea, and lethargy [75]. Chronic B toxicity is associated with symptoms including weight loss, reduced reproduction, and decreased appetite (Hunt 1993, cited in Nielsen [74]). However, for both humans and animals, no critical intake values have been described, which also applies to potentially B deficiency [e.g. 71]. Signs of B deficiency, however, are correlated with low immune function and increased mortality risk due to high incidence of osteoporosis [73].

Although it has been suggested that B is mostly accumulated in bone, nails and hair [e.g. 76, 77], we found it scattered throughout the whole body (Fig. 3a-h). The B concentration was the lowest for RD4 (Table 1), which tended to accumulate more in bone, eyes, intestines and stomach (Fig. 3d). B was not detectable in the bone and heart of RD3 but was present in any other tissue (Fig. 3c). Moreover, WB3 was the only individual with the highest B concentration in bone (Table 3; Fig. 3g), while the B concentration was notably the highest in the stomach and intestines for WB4 (Table 3; Fig. 3h).

### Calcium (Ca)

Ca has many functions in the animal body, including the formation of skeletal tissues, transmission of nervous tissue impulses, excitation of skeletal and cardiac muscle contraction, and blood clotting (e.g. 54, 78]. Excessive dietary intake is not associated with any specific signs of [54]. Ca toxicity—aka hypercalcemia—can manifest in many different forms, including renal stones, bone pain, gastrointestinal abdominal moans, neuromuscular psychic groans, and cardiovascular issues [e.g. 79]. Ca deficiency—aka hypocalcemia—is most dangerous for young animals, which leads to reduced mineralization of new bones and therefore reduced growth [54, 80]. Like a deficiency of vitamin D or P, a deficiency of Ca can also contribute to rickets [54, 80].

We found Ca in all body tissues for all individuals (Fig. 4a-h). NRC [54] described that about 98 percent of the total Ca pool is located in the bones. However, although we found the highest Ca concentration in bone for all individuals (Tables 2 and 3), we found that the Ca concentrations in bone contributed to 86 to 94 percent of the total Ca of deer (Tables 1 and 2), and 92 to 97 percent for boar (Tables 1 and 3). WB4 had the lowest Ca concentration in its body (Table 1), of which the concentration in bone was about 92 percent of the total Ca (Tables 1 and 3), and was the only boar with a total Ca concentration lower than 300,000 µg Kg^−1^ (Table 1). RD1 had the highest Ca concentration of the deer, followed by RD4 (Table 1). For these individuals, the percentage of Ca stored in bone was higher compared to RD2 and RD4 (Tables 1 and 2).

### Cadmium (Cd)

Cd is a highly toxic heavy metal that has no essential function in any physiological and biochemical process [e.g. 54, 81–85]. It accumulates in kidney, causing renal damage, from where it is very poorly and slowly excreted [54]. It is antagonistic to Zn, Cu and Fe [54, 86]. Acute Cd toxicity appears to be rare, but chronic Cd toxicity may be expressed by disturbed renal function, altered reproduction, and damaged lung function [e.g. 87].

For all individuals, we indeed found the highest Cd concentrations in the kidney, but we also detected it in liver and, for some individuals, in pancreas (Fig. 5a-h). In one wild boar (WB1), however, we found Cd more scattered throughout the body (Fig. 5e), which might be due to a lower Se concentration (Table 1) as Se can act as antioxidant for both As and Cd toxicity [88].

### Cobalt (Co)

Co is a scarce but essential trace element that is an important component of vitamin B12 [54]. Although Co toxicity is less plausible due to its scarcity, symptoms of Co toxicity include reduced feed intake, hyperchromemia and eventually anemia [e.g. 89, 90]. Co deficiency seems much more likely to occur and include symptoms as fatty degeneration of the liver, anemia with pale mucous membranes, and increased susceptibility to infections due to impaired neutrophil function [91–93].

Although ruminants seem to be more sensitive to Co deficiency than monogastric animals [54], we found higher Co concentrations for deer than for boar (Table 1). For boar, we found comparable concentrations as found by Gasparik et al. [94], although they only measured muscle, liver and kidney. For all deer, we found the highest concentrations in liver (Fig. 6a-d; Table 2), while the guts were more prominent for boar (Fig. 6e-h; Table 3). Overall, we found Co more scattered throughout the body than expected.

### Chromium (Cr)

Although some forms of Cr are known as ecotoxic metals, Cr is an important trace element for normal glucose metabolism, especially when animals experience physiologic stress [e.g. 54]. Cr toxicity is primarily linked to hexavalent Cr (Cr^6+^) exposure, that passes the cell walls faster and is at least five times more toxic than other Cr forms, eventually causing pathologic changes in the DNA [e.g. 54, 95–97]. Symptoms of Cr deficiency include reduced insulin sensitivity and reduced growth [e.g. 98]. Other symptoms may include glucose intolerance, hunger hyperglycemia, neuropathy, and reduced muscle proportion [e.g. 99, 100].

Gasparik et al. [29] reported Cr concentrations in liver, kidney, and muscle of Red deer in Slovakia, and found comparable Cr concentrations between these tissues. However, we found that these tissues did not store the major Cr pool in the body (Fig. 7a-h). Cr was more randomly scattered throughout the whole body for RD1, RD3, RD4, WB1, WB2, and WB4 (Fig. 7c-f, h). Cr was predominantly found in the intestines of RD2 and WB3 (Fig. 7b, g), the individuals with the highest Se concentrations (Table 1).

### Copper (Cu)

Cu is an essential element that is a component of many enzymes in a wide range of biochemical processes that include cellular respiration, free radical detoxification and iron transport [54]. Cu toxicity has been most described for ruminants, especially cattle [e.g. 54, 101]. Commonly described symptoms include lethargy, recumbency, pale mucous membranes, excessive thirst, and jaundice [e.g. 102]. Cu deficiency has been reported as ataxia in adult deer [e.g. 103–105]. For ungulates in general, Cu deficiency may manifest subclinical with low Cu concentrations in liver and serum but without any other signs of poor health [e.g. 106–108].

Traditionally, Cu concentrations are measured in the liver and kidney [e.g. 109]. Although we found the highest concentrations in liver for most of the deer (Table 2) and in kidney for some boar (Table 3), we found Cu present throughout the whole body (Fig. 8a-h). It has been described in cattle that Cu accumulates in the liver before toxicosis becomes evident [54]. This seems unlikely for the deer and boar that we measured due to the scarcity of Cu in nature [e.g. 110]. Furthermore, levels of Cu concentration that are considered as normal seem to be unclear or case specific as McCullough [111], for instance, reported Cu levels in liver between 84 and 142 ppm and considered these to be normal, which is up to 600 times higher than the concentrations we found (Tables 1, 2 and 3).

### Iron (Fe)

Fe functions as a component of heme in hemoglobin and myoglobin and is therefore very important for all vital organs in the body [54]. When Fe uptake exceeds the binding capacity, free Fe may increase in the body, which is very reactive and can cause increased free radical production and oxidative stress, which increases the need of anti-oxidants [112]. This is associated with symptoms like diarrhea, reduced feed intake and weight gain [54]. Fe levels in water are believed to be higher than those in food items and should not exceed 0.3 mg Fe per liter for humans, while animals may be able to cope with higher concentrations [54]. Fe deficiency seems to be more likely to occur, of which a major symptom is hypochromic microcytic anemia, which is the result of improper hemoglobin production [54]. Another symptom can be increased morbidity and mortality due to depressed immune responses [113]. Generally, Fe deficiency is very rare due to the ubiquitous nature of Fe in the environment including soil contamination, and requirements decrease with increase of age [91].

We found Fe in all body tissues that we measured (Fig. 9a-h). For both species, we found the highest concentrations in lungs—a vital organ (Reece et al. 2011)—and the lowest concentrations in bones (Tables 2 and 3). However, we cannot rule out that this is due to the gunshot.

### Potassium (K)

K is one of the most abundant elements in the body and is important for many life functions including maintaining osmotic pressure, acid–base regulation, nerve impulse transport and muscle contraction [54]. Under natural conditions, K toxicity seems unlikely to occur [54] and it is not well defined which dietary K concentration may lead to toxicity [114]. It has been suggested that K toxicity can cause cardiac arrest [115]. A daily intake of 0.06 to 0.15 percent K of the total food intake has been reported as too low for dairy cattle [116, 117]. Signs of K deficiency include reduced feed and water intake, weight loss, loss of hair flossiness, and decreased pliability of the skin [e.g. 54].

K is thus needed in the entire body and, as such, we found it in all tissues that we measured (Fig. 10a-h). For deer, spleen tended to store the highest concentrations of K (Table 2), while we did not identify a specific tissue for boar (Table 3). Bone, and skin and hair appeared to store the lowest K concentrations for both species (Tables 2 and 3).

### Magnesium (Mg)

Similar to K, Mg is an essential element that is needed for enzymatic reactions vital to every major metabolic pathway, normal nerve conduction, muscle function and bone mineral formation [54]. Animals may suffer from skeletal abnormalities when they consume excessive amounts of Mg in their diets [e.g. 118], which is unlikely to occur since most animals are able to excrete large amounts of Mg via urine [54]. Symptoms of Mg deficiency—which is often described for livestock, despite being the fourth most abundant cation in the world—have been extensively described and include muscle twitches, tremors, osteoporosis, and cramps [e.g. 119–121].

We found Mg throughout the whole body, with no particular target tissue (Fig. 11a-h). Mg was detectable in all tissues, except the intestines of RD3 (Fig. 11d), which we would attribute to a measurement error since this is the only missing Mg concentration in our data. We found rather similar Mg concentrations for deer and boar, ranging from 19,319 to 36,420 µg Kg^−1^ (Table 1).

### Manganese (Mn)

Mn is an essential trace element that is important for the forming of connective tissue, bones, blood clotting and sex hormones [54]. Mn toxicity has been widely described [e.g. 122, 123], and can be associated with many symptoms including Parkinsonism, bradykinesia, tremor, impaired postural reflexes and dystonia [123, 124]. Other symptoms include, especially for ruminants, reduced food intake and growth [125]. It has been shown that rats and humans that suffered from Fe deficiency experienced increased Mn absorption [e.g. 126], and vice versa [e.g. 127, 128]. Although Mn deficiency is unlikely to occur since Mn is available in nearly all food items [129], Mn deficiency reveals most likely in the form of skeletal abnormalities [e.g. 123, 130], like enlarged joints, deformed legs with thickened and shortened long bones, and overall lameness in pigs, ruminants and poultry [131].

Mentioned target tissues for Mn include skeleton, liver and hair [132], or brain and bone [e.g. 133–135]. Mn has been traditionally measured in tissues as liver, kidney and muscle [e.g. 29, 30]. We found the highest concentrations in the guts for most individuals (Fig. 12a-h). We found the highest concentration in bone only for WB3 (Tables 1 and 3; Fig. 12g). Bone stored the least Mn for most of the other individuals (Tables 2 and 3). We found overall higher Mn concentrations for deer than for boar, respectively ranging from 2,785 to 5,462 µg Kg^−1^ and from 698 to 1,902 µg Kg^−1^ (Table 1).

### Molybdenum (Mo)

As an essential trace element, Mo is a component of many enzymes throughout the body, including enzymes found in milk (Mills and Davis 1987, cited in [54]). Mo is antagonistic to Cu, implying that Mo toxicity can occur in the form of Cu deficiency [e.g. 54, 136, 137]. Ruminants would be more sensitive to Mo toxicity than monogastric animals [137]. Mo toxicity can cause diarrhea, anorexia, depigmentation of hair, neurological disturbances and premature death [138]. Although naturally occurring Mo deficiency has never been demonstrated in free-living animals, Mo deficiency can be the result of low Mo levels in soil, plants, drinking water and other food items [e.g. 139]. Mo is known for its anticarcinogen properties, low concentrations being associated with oesophageal cancer in particular [e.g. 139–141].

Mo concentrations are traditionally measured in liver and kidney [e.g. 136, 139, 142]. However, we found no specific tissue that stored the majority of the Mo pool in the body (Fig. 13a-h). The eyes turned out to have the major content for WB3 (Fig. 13g), while Mo had been hardly detected in this tissue for most of the other individuals (Fig. 13a-d, f, h). We found slightly higher Mo concentrations in deer than in boar, ranging from 47.30 to 59.71 µg Kg^−1^ and 35.63 to 59.42 µg Kg^−1^, respectively (Table 1).

### Sodium (Na)

Na is an essential macro element that is important for life functions including controlling blood pressure, blood volume and water balance [54]. A proper Na and K balance is required for heart function and nerve impulse conduction [e.g. 54], and it is a major component of salts in saliva to buffer acid from ruminal fermentation [143]. When the Na concentration in the blood is too high, which can be a result of dehydration, animals can suffer from hypernatremia, manifesting in symptoms like excessive thirst or lethargy [e.g. 144, 145]. Excessive Na levels would be first detectable in the brain [e.g. 146, 147]. Animals that suffer from Na deficiency are described to have an intense craving for salt, that they show by chewing and licking various objects [e.g. 54, 148].

We detected Na in all tissues that we measured (Fig. 14a-h). The highest Na concentrations were found in the eyes of boar (Table 3), and in the eyes and brain of deer (Table 2). We found the lowest concentrations in muscle, skin and hair (Tables 2 and 3). Overall, the deer tented to have slightly higher Na concentrations compared to the boar, ranging from 145,511 to 156,760 µg Kg^−1^ and 117,545 to 158,860 µg Kg^−1^, respectively (Table 1).

### Nickel (Ni)

The best described functions of the essential trace element Ni include increasing hormonal activity, lipid metabolism, and urease activity [e.g. 12, 54, 149]. One of the best described forms for Ni toxicity is found in its carcinogenic effects [e.g. 150, 151]. Other toxic effects are genotoxic, immunological, endocrine, neurogenic, cardiovascular, gastrointestinal, musculoskeletal, dermal and metabolic [150], although it remains unclear when Ni accumulation would become a problem for wildlife [152]. Naturally occurring Ni deficiency is rare due to the extremely low intake requirements [153–155]. Signs of Ni deficiency include depressed growth, lower reproduction, lower plasma glucose, or altered distribution of e.g. Fe, Cu, Ca, and Zn [156].

Although it has been suggested that Ni is, once ingested, distributed mostly to kidney, bone and lungs [e.g. 54], we found it more randomly throughout the body (Fig. 15a-h). For some individuals—RD2, RD3, RD4, WB2, and WB3—we indeed found relatively high concentrations in lungs (Tables 2 and 3; Fig. 15b-d, f-g). We found remarkably high concentrations of Ni in the pancreas of RD1 (Table 2; Fig. 15a), the intestines of RD2 and WB1 (Tables 2 and 3; Fig. 15b, e), and the spleen of WB4 (Table 3; Fig. 15h).

### Phosphorous (P)

P has more known functions in the animal body than any other element and is located in all body cells where it is involved in nearly all energy transactions [e.g. 54, 119]. When P is excessive in the diet for a long period, it can cause problems of Ca metabolism [e.g. 54]. This is most likely to occur in monogastric animals since ruminants can tolerate a wider Ca:P ratio [54]. P deficiency seems most likely to occur when animals forage on P poor soils [e.g. 157, 158]. General signs of P deficiency include weight loss, stiff joints and muscular weakness [e.g. 54, 159]. Other symptoms can be the desire to eat wood, bones, rocks and other materials [e.g. 160, 161].

We found P in every tissue and none of the tissues in particular (Fig. 16a-h). Most individuals had the highest P concentrations in bone and the lowest concentrations in skin and hair, or eyes (Tables 2 and 3). We found similar concentrations for deer and boar, ranging from 273,821 to 376,183 µg Kg^−1^ and from 223,948 to 399,327 µg Kg^−1^, respectively (Table 1).

### Lead (Pb)

Pb is the most common cause of toxicoses in animals [162]. There is no evidence for its essentiality [e.g. 54, 162]. Often described symptoms of Pb toxicity include disturbed muscular coordination, reduced cognitive performance and anemia [e.g. 162, 163]. Pb disturbs the balance between functions of other metals including Cu and Zn [164].

Accordingly other heavy metals, the liver and kidney are traditionally considered as the target organs for Pb [e.g. 30, 164, 165]. It is also believed to accumulate in bone [e.g. 86, 166]. We found bone as the major storage pool of Pb for RD1, WB1, WB2 and WB3 (Fig. 17a, e–g), whereas we found the highest concentrations in the digestive system for RD2 and WB4 (Fig. 17b, h). For RD4 and WB3, Pb was more scattered throughout the body (Fig. 17d, g), which was also the case to a lesser extent for WB1 (Fig. 17e). RD3 was the only individual with the highest Pb concentration in the lungs (Fig. 17c), with considerably higher concentrations compared to any of the other individuals (Tables 1, 2 and 3). This might be a sign of toxicosis, as the lungs are one of the vital organs [52], although reference values are missing to validate this presumption.

### Sulfur (S)

S is an essential element that has many functions in the animal body, including forming several amino acids, and promoting DNA fixation and the antioxidant systems [e.g. 54, 167]. It is present in every body cell [e.g. 168]. S toxicity is most likely to manifest neurologic transmissions, causing acute symptoms including blindness, muscle twitches and recumbency [169]. Other symptoms include severe enteritis, peritoneal effusion, and petechial hemorrhages in especially kidney [170]. It may occur that S toxicity can be smelled in the breath [54]. Symptoms of S deficiency in ruminants include reduced food intake, weight and hair loss, overall weakness and death, which are all signs of digestive tract or metabolism problems [168, 171]. Since S deficiency has been mostly studied in ruminants, it is unclear whether monogastric animals experience similar symptoms.

Kierdorf et al. [172] found S in higher concentrations in deer’s antlers compared to pedicles. We found overall slightly higher S concentrations in deer compared to boar (Table 1). Skin and hair seems to contain the highest S concentrations for deer (Table 2), while we did not find any specific tissue for boar (Table 3). As S is part of every body cell, we found it distributed throughout the whole body with no particular target tissue (Fig. 18a–h).

### Selenium (Se)

Se has antioxidant properties when supplied in low concentrations [e.g. 88]. It is important in several enzymes, helps to make DNA, and protects against cell damage and infections (e.g. [54]). The soil is the best Se source for all life forms, although the Se concentration in the soil does not seem to be the best indicator of Se availability for animals [173, 174]. Se toxicity is most commonly known in the form of alkali disease—aka selenosis, severely damaged hooves [e.g. 54, 174]. Se deficiency can cause white muscle disease or nutritional muscular dystrophy [e.g. 54], often resulting in death.

Kidney or liver are most commonly used as bioindicator of Se in the environment [e.g. 38, 39, 175, 176]. We found the highest Se concentration in kidney for all individuals (Tables 2 and 3), while it was also found in nearly all other tissues that we analyzed in lower concentrations (Tables 2 and 3; Fig. 19a-h). We found the highest concentrations in boar and the lowest concentrations in deer (Table 1).

### Silicon (Si)

Si is only found in very trace amounts in animal bodies [54]. It has a role in connective tissue and healing from injuries [e.g. 54, 55, 177, 178]. Since Si is easily excreted, Si toxicity is unlikely to occur [179]. Si deficiency may lead to delays in growth, bone deformations and abnormal skeletal development [177]. It affects connective tissue metabolism and thus organic bone formation [180].

Although we found a lot of variation in the total Si concentration among individuals (Table 1) and it was scattered throughout all tissues we measured (Fig. 20a-h), Si slightly tended to be most concentrated in the digestive system—intestines or pancreas—for some individuals (Tables 2 and 3). Moreover, Bellés et al. [181] described that Si prevents Al absorption and reduces Al concentrations in tissues including brain, liver, bone, kidney, and spleen. We found, however, the highest Al concentrations in the individuals with the highest Si concentrations—RD2, RD4, and WB4 -, while for none of these individuals the highest Si concentrations were measured in brain, liver, bone, kidney, or spleen (Fig. 1b, d, h; Tables 1, 2 and 3).

### Strontium (Sr)

Sr can be seen as the chemical analog of Ca, and as such, its major role is found in the formation and breakdown of bony material [e.g. 182–184]. Bony material is most commonly used to measure the Sr concentration in animals [185]. Sr toxicity seems unlikely to occur since dietary Sr can vary widely without any toxic symptoms [186]. It has been mentioned that high dietary Sr increased the risk of P deficiency [187]. Although Sr has never been shown to be an essential element, Sr has been shown to promote bone Ca and to reduce fracture rate in osteoporotic patients [186]. This might imply that osteoporosis may be related to Sr deficiency, although reference values to investigate this presumption are missing [186].

We found indeed the highest Sr concentrations in the bones of all individuals (Tables 2 and 3; Fig. 21a-h). Skibniewski et al. [188] used muscle as model tissue, however muscle belonged to the tissues with lowest Sr concentration in our analysis (Fig. 21a-h), which was for RD3 and RD4 even the tissue with the lowest Sr concentration (Table 2).

### Zinc (Zn)

As a component of many enzymes, Zn affects the metabolism of carbohydrates, proteins, lipids, and nucleic acids, and it helps in regulating hormones and the immune system [e.g. 12, 54, 189]. Zn salts have been shown to protect against different forms of toxicity, including Cu toxicity [190–192]. Zn toxicity is most likely to occur in the form of Cu deficiency since Zn and Cu are antagonistic to each other [54, 193]. Excessive Zn uptake can also give symptoms including epigastric pain, lethargy, and fatigue [194]. Zn deficiency include symptoms as reduced feed intake and reduced growth [54].

Although most studies measure Zn in the liver, kidney, or muscle (e.g. 24, 175, 188, 189), we found Zn scattered throughout the whole body, with no particular tissue as major storage pool (Fig. 22a–h). Overall, deer tended to have slightly higher Zn concentrations compared to boar (Table 1).

## Discussion and Conclusions

In this study, we aimed to gain insights in the ionome of large mammals, by measuring 22 chemical elements across 13 tissues of two ungulate species (deer and boar), and evaluating how these elements are distributed over the body. We used four individuals of deer and boar that lived in a nutrient-poor Dutch National Park (Veluwezoom), where deficiencies are most likely to occur. We found that de ionome was highly variable between and within the two species. For most elements, tissues having the highest and lowest concentration differed between individuals (Tables 2 and 3). No single tissue accurately represented the accumulation of toxic or scarce elements in the bodies. These findings imply that analyses of elemental concentrations in single tissues do not necessarily reflect bioaccumulation of toxins or deficiencies of scarce elements.

We attempted to put our findings in the context of the biological and physiological role of the elements, and noticed that the lack of reference values per element per species indeed limited our understanding and the interpretation of the measured concentrations. Such reference values are required to determine any toxicities or deficiencies. The signs of toxicities or deficiencies are element-specific [e.g. 39, 195–197], and can be even species-specific [e.g. 197–199]. Since the margins per element for wild deer and boar remain unknown, we were unable to assess toxicities or deficiencies. Therefore, the elemental concentrations that we reported here must be seen as a first step to enlarge the comprehension of the elemental composition of wild living deer and boar.

Our study showed that at the individual level most elements are rather scattered throughout the whole body. Also elements that seem to have a target tissue, e.g. kidney for Se (Fig. 19a-h), appeared to be more scattered than expected. Thus, when focusing on only a few samples of tissues traditionally mentioned as storage pools for particular elements, there is a high risk of underestimation or missing crucial information, which may lead to wrong conclusions. Moreover, we encourage further studies to collect samples of individuals in the shortest time interval, largest sample sizes, and most complete ionomes as possible.

We see four possible limitations of our study. First, the sample size per species was low given the high variability of elemental concentrations that we found within and between the species. Many more individuals would probably be needed to attain stable averages, if possible, of elemental concentrations. Second, the individuals we dissected were obtained from regular culling. This might introduce some unintended bias due to potential harvest bias in terms of sex, age and condition [e.g. 40, 200, 201]. Third, salt licks and corn—for the deer and boar, respectively—were provided occasionally to facilitate culling. Although we measured these salt licks and corn for the same elements as we did for the tissue samples that we analyzed (Appendix 2), we are only able to speculate about the effect of these supplements on the elemental concentrations we that found. For instance, the mineral licks contained more Co and Cu than the corn (Appendix 2). Since we found higher Co and Cu concentrations in deer compared to boar (Table 1), it might be possible that the mineral licks contributed to these higher concentrations, although any evidence is missing [e.g. 135]. We assume that these supplements attracted the individuals equally. Fourth, we did not include the antlers of male deer in our analysis. Therefore, we cannot address their potential function of yearly shedding in reducing the ecotoxic burden in male deer. We propose to include this as an extra tissue in future studies examining the full ionome of deer. We do not believe, however, that any of the above-mentioned limitations affect the conclusions of our study.

We encourage other scientists to execute extensive surveys of the elemental composition of wild living animals, including as much information possible about these animals and the circumstances they encountered during their lives. This will not only improve the physiological understanding of trace elements in the animal body, but also enables us to link ionomic insights to ecological processes. We propose to install an international database where all the measured elemental concentrations can be uploaded per tissue and species combination, including the sex, status (e.g. pregnancy, lactating) and age of the individuals, to enlarge the current knowledge and to potentially approach reference values in the future.

### Supplementary Information

Below is the link to the electronic supplementary material.

Supplementary file1 Letter of approval of the Animal Welfare Centre. (PDF 67 KB)

Supplementary file2 (DOCX 16 KB)

## Data Availability

The complete dataset will be accessible through Figshare: https://doi.org/10.6084/m9.figshare.23633997.
