# Supplementary material for: Variability of the Ionome of Wild Boar (Sus scrofa) and Red Deer (Cervus elaphus) in a Dutch National Park, with Implications for Biomonitoring
Source: Biol Trace Elem Res. 2023 Oct 9;202(6):2518–46. doi: 10.1007/s12011-023-03879-7 (PMC11052835; doi:10.1007/s12011-023-03879-7)
Supplement: Supplementary file 1 — Supplementary file1 Letter of approval of the Animal Welfare Centre. (PDF 67 KB) [file 12011_2023_3879_MOESM1_ESM.pdf]

WAGENINGEN UNIVERSITY
WAGENINGEN UR

For quality of life

Memo

Corporate Education and Research

TO
Elke Wenting

FROM
Janneke Arts, Animal Welfare Officer

DATE
20/01/2023

POSTAL ADDRESS
Bode 120
The Netherlands

VISITORS' ADDRESS
Bornse Weilanden 5
6708 WG Wageningen

INTERNET
www.wageningenuniversity.nl

HANDLED BY

TELEPHONE

EMAIL

Dear Elke,

The Animal Welfare Body assessed the work protocol**: "Variability of the ionome of Wild boar (Sus scrofa) and Red deer (Cervus elaphus) in a Dutch national park, with implications for biomonitoring**, received on **16/01/2023**.

It is the opinion of the AWO that this is not an animal experiment as referred to in the Dutch Act on Animal Experiments, since the experimental procedures described in present protocol will not make use of live animals or animals that were killed for the purpose of tissue sampling.

In any unforeseen circumstances, or intended deviations from the aforementioned protocol, please contact the animal welfare officer.

Yours sincerely,
Dr. Ir. Ing. J.W.M. Arts

Animal Welfare Officer

Wageningen UR (Wageningen University and various research institutes) is specialised in the domain of healthy food and living environment.
