# Supplementary material for: Variability of the Ionome of Wild Boar (Sus scrofa) and Red Deer (Cervus elaphus) in a Dutch National Park, with Implications for Biomonitoring
Source: Biol Trace Elem Res. 2023 Oct 9;202(6):2518–46. doi: 10.1007/s12011-023-03879-7 (PMC11052835; doi:10.1007/s12011-023-03879-7)
Supplement: Supplementary file 2 — Supplementary file2 (DOCX 16 KB) [file 12011_2023_3879_MOESM2_ESM.docx]

|  | Corn | Salt lick 1 | Salt lick 2 |
| --- | --- | --- | --- |
| Al | - | - | 1.79 |
| As | 0.024 | - | 0.0375 |
| B | 4.65 | - | - |
| Ca | 67.15 | - | - |
| Cd | 0.0089 | - | - |
| Co | - | 0.0157 | 0.153 |
| Cr | - | - | - |
| Cu | 5.29 | 338 | 2842 |
| Fe | 18.29 | - | 1,067 |
| K | 8,259 | - | - |
| Mg | 2,695 | 1,541 | 1,527 |
| Mn | 20.29 | - | 129 |
| Mo | 0.235 | - | 0.217 |
| Na | - | 658,178 | 640,362 |
| Ni | 0.549 | 1.907 | 1.402 |
| P | 7429 | - | - |
| Pb | - | - | - |
| S | 2,013 | - | - |
| Se | 0.021 | 35.0 | 16.561 |
| Si | 10.23 | 37.33 | 3.667 |
| Sr | - | 0.159 | 0.319 |
| Zn | 43.93 | 1,255 | 519 |

Appendix B Elemental concentrations (µg Kg^-1^) of the salt licks - for deer - and corn - for boar - used to lure animals to cull sites in Veluwezoom National Park.
